# Exploration of the Nuclear Proteomes in the Ciliate *Oxytricha trifallax*

**DOI:** 10.3390/microorganisms11020343

**Published:** 2023-01-30

**Authors:** Michael W. Lu, Leslie Y. Beh, V. Talya Yerlici, Wenwen Fang, Katarzyna Kulej, Benjamin A. Garcia, Laura F. Landweber

**Affiliations:** 1 Department of Biological Sciences, Columbia University, New York, NY 10025, USA; 2 Department of Biochemistry and Molecular Biophysics, Columbia University, New York, NY 10032, USA; 3 RNA Therapeutics Institute, UMass Chan Medical School, Worcester, MA 01655, USA; 4 Division of Protective Immunity and Division of Cancer Pathobiology, The Children’s Hospital of Philadelphia, Philadelphia, PA 19104, USA; 5 Department of Biochemistry and Molecular Biophysics, Washington University in St. Louis, St. Louis, MO 63130, USA

**Keywords:** *Oxytricha*, nuclear proteome, ciliate, proteomics, macronucleus, micronucleus, histone

## Abstract

Nuclear dimorphism is a fundamental feature of ciliated protozoa, which have separate somatic and germline genomes in two distinct organelles within a single cell. The transcriptionally active somatic genome, contained within the physically larger macronucleus, is both structurally and functionally different from the silent germline genome housed in the smaller micronucleus. This difference in genome architecture is particularly exaggerated in *Oxytricha trifallax*, in which the somatic genome comprises tens of thousands of gene-sized nanochromosomes maintained at a high and variable ploidy, while the germline has a diploid set of megabase-scale chromosomes. To examine the compositional differences between the nuclear structures housing the genomes, we performed a proteomic survey of both types of nuclei and of macronuclear histones using quantitative mass spectrometry. We note distinct differences between the somatic and germline nuclei, with many functional proteins being highly enriched in one of the two nuclei. To validate our conclusions and the efficacy of nuclear separation, we used protein localization through a combination of transformations and immunofluorescence. We also note that the macronuclear histones strikingly display only activating marks, consistent with the conclusion that the macronucleus is the hub of transcription. These observations suggest that the compartmentalization of different genome features into separate structures has been accompanied by a similar specialization of nuclear components that maintain and facilitate the functions of the genomes specific to each nucleus.

## 1. Introduction

The nucleus is an integral component of the eukaryotic cell and plays an important role in many cellular processes, such as DNA replication, repair, transcription, and reproduction. Its myriad functions are facilitated by the complex composition of the nucleus, which includes a wide range of proteins and nucleic acids [1]. These components interact with each other to form the requisite structures and complexes for performing nuclear functions in several different suborganellar compartments, including ribosomal biogenesis within the nucleolus, nuclear import and export through nuclear pore complexes, and transcriptional regulation at compartments of active chromatin [2,3].

Ciliates are a phylum of unicellular eukaryotes that share the unique feature of housing two different genomes within two separate types of nuclei in a single cell. While metazoans evolved a separation between the soma and germline by differentiation of cell types, ciliates achieve this distinction between germline and soma by maintaining distinct types of nuclei within a single cell. The differences between the somatic and germline genome in ciliates are also far greater than in other eukaryotes. The somatic genome, contained in the physically larger macronucleus (MAC), is comprised of small polyploid chromosomes that contain primarily coding sequences that are actively transcribed throughout the asexual (vegetative) life cycle. The germline genome is housed in the physically smaller micronucleus (MIC) and is generally more structurally similar to a typical eukaryotic genome. However, it is transcriptionally inactive in vegetative cells and only becomes active during the process of sexual conjugation, during which two cells of compatible mating types exchange haploid copies of their MIC that subsequently fuse, and a copy of the new zygotic MIC undergoes a complex series of genome rearrangements to produce a new MAC [4,5].

The differences between the MAC and MIC genomes are particularly exaggerated in the hypotrichous ciliate *Oxytricha trifallax*. The MAC genome of this species comprises over 18,000 distinct gene-sized “nanochromosomes” averaging just 3 kb in length, most of which encode only a single gene. These nanochromosomes are also maintained at variable ploidy, with multiple orders of magnitude difference between the most and least abundant nanochromosomes [6,7]. These drastic differences in genome organization between the MAC and MIC genomes of *Oxytricha* suggest that a similar disparity between the composition of the nuclei, themselves, may exist in order to facilitate the different functions and features of the two genomes.

Previous studies in other ciliates have identified many compositional differences between the two types of nuclei. These studies have focused primarily on small subsets of nuclear features, such as histone composition and modifications [8,9], nuclear pore complexes [10], or karyopherins [11]. These studies have reported that despite containing similar structures, the corresponding components of these complexes may differ, with alternative variants present between the MAC and MIC versions for some proteins, while the two nuclei share the same versions of other proteins. To our knowledge, there have been no published reports that provide a comprehensive description and comparison of the compositions of the MAC and MIC proteomes of a ciliate.

To address this limitation in our current understanding of ciliate biology, here we used a quantitative mass spectrometry approach utilizing isobaric labeling to describe the MAC and MIC proteomes of *Oxytricha*. We identified over 3600 proteins, the majority of which are likely nuclear. 285 of these proteins are significantly enriched within the MAC, and 552 are significantly enriched in MIC samples, although many additional proteins are likely nucleus-specific as well. We also performed a rigorous analysis of the MAC histones and their modifications. We identified many groups of proteins associated with known features of the two nuclei, confirming differences in their composition and providing a reference for further investigation of the unique features of ciliate nuclei.

## 2. Materials and Methods

### 2.1. Cell Culture

*Oxytricha trifallax* strain JRB310 was cultured vegetatively at a concentration of ~15,000 cells/mL in Pringsheim media (0.11 mM Na_2_HPO_4_, 0.08 mM MgSO_4_, 0.85 mM Ca(NO_3_)_2_, 0.35 mM KCl, pH 7.0) and fed daily with *Chlamydomonas reinhardtii*, supplemented with 1:1000 culture volume of *Klebsiella pneumoniae* overnight liquid culture every other day. Cells were filtered through cheesecloth daily to remove debris [12]. 

### 2.2. Nuclear Enrichment

Cells were starved for 2–3 days at 4 °C to allow for the digestion of previously consumed food while preventing encystment. After starvation, cells were collected on a 10μm Nitex mesh filter and concentrated by centrifugation at 200× *g* for 1 min. Following removal of the supernatant, cells were lysed in Lysis buffer (20 mM Tris pH 6.8, 3% *w/v* sucrose, 0.2% *w/v* Triton X-100, 0.01% *w/v* spermidine trihydrochloride) for 30 minutes on ice with occasional vortexing. After visually confirming the complete lysis of whole cells through DAPI staining and fluorescence microscopy, the MAC and MIC were separated using 10%–40% discontinuous sucrose gradients. A layer of 10% sucrose in TSC (20 mM Tris pH 6.8, 0.1% Triton X-100, 0.01% *w/v* spermidine trihydrochloride, 5 mM CaCl_2_) was placed over a layer of 40% sucrose in TSC and cell lysate was layered on top of the gradient. After centrifugation at 250× *g* for 10 min in a Sorvall SH-3000 the 10% layer was collected as the MIC fraction and the 40% layer along with the pellet were collected as the MAC fraction [13]. The resulting nuclear fractions were pelleted by centrifugation at 4700 rpm for 10 min and frozen in LN_2_, then stored at −80 °C. This procedure was performed 3 times, using 20 million cells as input for each sample.

### 2.3. Mass Spectrometry of Separated Nuclear Samples

For global quantitative proteomics analysis of nuclear samples, tandem mass tag (TMT)-based quantitative proteomics was used [14]. In brief, frozen nuclei were lysed by bead-beating in 8 M urea and 200 mM EPPS (pH 8.5), supplemented with protease inhibitors. Samples were reduced with 5 mM TCEP and alkylated with 10 mM iodoacetamide (IAA) that was quenched with 10 mM DTT. A total of 200 μg of protein was chloroform−methanol precipitated. Protein was reconstituted in 200 mM EPPS (pH 8.5) and digested by Lys-C overnight and trypsin for 6 h, both at a 1:50 protease-to-peptide ratio. Digested peptides were quantified using a Nanodrop at 280 nm and 100 µg of the peptide from each sample were labeled with 800 µg TMT reagent using 10-plex TMT kit. TMT labels were checked, 0.5 µg of each sample was pooled, desalted, and analyzed by short SPS-MS3 method, and using normalization factor, samples were bulk mixed at 1:1 across all channels. Mixed TMT-labeled samples were vacuum centrifuged and fractionated using the Pierce™ High pH Reversed-Phase Peptide Fractionation Kit (Thermo Scientific, Waltham, MA, USA) and each fraction was dried down in a speed-vac. Dried peptide were dissolved in 10 µL of 3% acetonitrile/0.1% formic acid injected using SPS-MS3. The UltiMate™ 3000 RSLCnano system (Thermo Scientific) and EASY Spray™ source (Thermo Scientific) with Acclaim™ PepMap™100 2 cm × 75 μm trap column (Thermo Scientific) and EASY-Spray™ PepMap™ RSLC C18 50 cm × 75 μm ID column (Thermo Scientific) were used to separate fractioned peptides with a 5–30% acetonitrile gradient in 0.1% formic acid over 127 min at a flow rate of 250 nL/min. After each gradient, the column was washed with 90% buffer B for 5 min and re equilibrated with 98% buffer A (0.1% formic acid, 100% HPLC-grade water) for 40 min. For BPRPseparated proteome fractions, the full MS spectra were acquired in the Orbitrap Fusion™ Tribrid™ Mass Spectrometer (Thermo Scientific) at a resolution of 120,000. The 10 most intense MS1 ions were selected for MS2 analysis. The isolation width was set at 0.7 Da and isolated precursors were fragmented by collision-induced dissociation (CID) at a normalized collision energy (NCE) of 35% and analyzed in the ion trap using “turbo” scan speed. Following acquisition of each MS2 spectrum, a synchronous precursor selection (SPS) MS3 scan was collected on the top 10 most intense ions in the MS2 spectrum. SPS-MS3 precursors were fragmented by higher energy collision-induced dissociation (HCD) at an NCE of 65% and analyzed using the Orbitrap.

### 2.4. Analysis of Nuclear Mass Spectrometry Results

The raw LC-MS/MS files were analyzed using MaxQuant [15] version 1.6.15.0 using the *Oxytricha* MAC protein database [7], along with the *Chlamydomonas reinhardtii*(UP000006906) and *Klebsiella pneumoniae*(UP000000265) UniProt databases to identify proteins from known potential contaminants. All fractions were grouped together and TMT 10plex labeling with quantification of reporter ions at the MS3 level was used. Methionine oxidation and N-terminal acetylation were set as variable modifications and carbamidomethylation was set as a fixed modification. Trypsin/P was used as the digestion enzyme with up to 2 missed cleavages. The precursor mass tolerances were set to 20 ppm for the first search and 4.5 ppm for the main search. A maximum FDR of 1% was applied at both the peptide and protein level for identification. Data from MaxQuant output was analyzed with R [16] using the RStudio IDE [17]. The TMT reporter ion intensities for each channel were normalized so the total sum of intensities for each channel equaled 1,000,000. Protein groups without any *Oxytricha* proteins were removed. Since the *Oxytricha* database used in this study contains many duplicated protein sequences due to the presence of nanochromosome isoforms and variants that encode identical proteins, the first entry of the majority protein was used as the representative protein in each protein group.

### 2.5. Quantitative Mass Spectrometry Analysis of Oxytricha Histone PTMs 

For bottom-up MS analysis, *Oxytricha* histones were acid-extracted from purified nuclei which were pelleted by centrifugation at 4000× *g*, resuspended in 0.421 mL 0.4 N H_2_SO_4_ per 10^6^ input cells, and nutated for 3 h at 4 °C to extract histones. Subsequently, the acid-extracted mixture was centrifuged at 21,000× *g* for 15 min to remove debris. Proteins were precipitated from the cleared supernatant using trichloroacetic acid (TCA), washed with cold acetone, then dried and resuspended in 2.5% (*v/v*) acetic acid. Individual core histone fractions were purified from crude acid-extracts using semi-preparative RP-HPLC (Vydac C18, 12 micron, 10 mM × 250 mm) with 40–65% HPLC solvent B over 50 min and dissolved in 30 μL of 50 mM NH_4_HCO_3_, pH 8.0. Derivatization reagent was prepared by mixing propionic anhydride with acetonitrile in a ratio of 1:3 (*v/v*), and such reagent was mixed with the histone sample in the ratio of 1:4 (*v/v*) for 15 minutes at 37 °C. This reaction was performed twice. Histones were then digested with trypsin (enzyme:sample ratio 1:20, overnight, room temperature) in 50 mM NH_4_HCO_3_. After digestion, the derivatization reaction was performed again twice to derivatize peptide N-termini. Samples were desalted prior to nLC-MS/MS analysis by using C18 Stage-tips. Samples were analyzed by using a nLC-MS/MS setup. Chromatography was configured with the same type of column and HPLC as for the proteomics analysis. The HPLC gradient was as follows: 2% to 28% solvent B (A = 0.1% formic acid; B = 95% MeCN, 0.1% formic acid) over 45 minutes, from 28% to 80% solvent B in 5 minutes, 80% B for 10 minutes at a flow-rate of 300 nL/min. nanoLC was coupled to an LTQ-Orbitrap Elite mass spectrometer (Thermo Scientific, San Jose, CA, USA). A full scan MS spectrum (*m/z* 300−1100) was acquired in the Orbitrap with a resolution of 120,000 (at 200 *m/z*) and an AGC target of 5 × 10^5^. MS/MS was performed using a data-independent acquisition (DIA) mode; the entire mass range (300–1100 *m/z*) was fragmented at every cycle using windows of 50 *m/z* (16 MS/MS scans total). MS/MS AGC target was 3 × 10^4^, the injection time limit was 50 msec and the CID collision energy was 35. MS/MS data were collected in centroid mode. EpiProfile was used to retrieve the extracted ion chromatograms and estimate the relative abundance of each peptide as compared to the total respective histone [18]. Histone protein sequence list was retrieved from the OxyDB database (http://oxy.ciliate.org/index.php/home (accessed on 1 February 2015)).

Middle-down mass spectrometry analysis was performed as follows: GluC was added to the histone sample at an enzyme:sample ratio of 1:20 (overnight digestion at room temperature). Reaction was blocked by adding 1% formic acid for LC-MS analysis. 4 biological replicates were used for the analysis. Samples were separated using an Eksigent 2D+ nanoUHPLC (Eksigent, part of ABSciex). The nanoLC was equipped with a two column setup, a 2 cm pre-column (100 µm ID) packed with C18 bulk material (ReproSil, Pur C18AQ 3 µm; Dr. Maisch, Ammerbuch, Germany) and a 12 cm analytical column (75 μm ID) packed with Polycat A resin (PolyLC, Columbia, MD, 1.9 µm particles, 1000 Å). Loading buffer was 0.1% formic acid (Merck Millipore, Burlington, MA, USA) in water. Buffer A and B were prepared as previously described [19]. The gradient was delivered as follows: 5 min 100% buffer A, followed by a not linear gradient from 55 to 85% buffer B in 120 min and 85–100% in 10 min. Flowrate for the analysis was set to 250 nL/min. MS acquisition was performed in an Orbitrap Fusion (Thermo) with a spray voltage of 2.3 kV and a capillary temperature of 275 °C. Data acquisition was performed in the Orbitrap for both precursor and product ions, with a mass resolution of 60,000 for MS and 30,000 for MS/MS. MS acquisition window was set at 660–740 *m/z*. Dynamic exclusion was disabled. Precursor charges accepted for MS/MS fragmentation were 5–8. Isolation width was set at 2 *m/z*. The 5 most intense ions with MS signal higher than 5000 counts were isolated for fragmentation using electron-transfer dissociation (ETD) with an activation time of 20 msec. 3 microscans were used for each MS/MS spectrum, and the AGC target was set to 2 × 10^5^. Data processing was performed as previously described [19]. Briefly, spectra were deconvoluted with Xtract (Thermo) and searched with Mascot (v2.5, Matrix Science, London, UK), including acetylation (K) as dynamic modifications. No fixed modifications were selected. Histone protein sequence list was retrieved from the OxyDB database (http://oxy.ciliate.org/index.php/home). Enzyme was GluC (cleaves after E) with 0 missed cleavages allowed. Mass tolerance was set to 2.1 Da for precursor mass and 0.01 Da for product mass. Mascot result files were processed using a tolerance of 30 ppm, as we previously demonstrated it is a suitable value to filter confident identification and quantification [19]. Peptides with ambiguous modification site assignments were automatically discarded by the software.

Middle-down and bottom-up MS-analysis were performed with four biological replicates (n_b_ = 4). Moreover, each biological replicate of the histone sample was performed in technical triplicates (n_t_ = 3) (with the exception of “Replicate 4” for middle-down data, where n_t_ = 2). No samples were excluded as outliers (this applies to all proteomics analyses described in this manuscript).

### 2.6. Protein Predictions

All proteins identified from the nuclear mass spectrometry results were used as input for software to analyze and predict features. InterProScan [20] version 5.45-80.0 was used to predict protein features using all available analyses, as well as GO terms and Pathway annotations. The Pannzer2 web server [21] was also used to predict GO terms. DeepLoc 2.0 [22] was used to predict subcellular localizations and sorting signals. The GO-MWU [23] R package was used to identify GO categories which had significant enrichment of genes predominantly found in either the MAC or MIC. BLASTP [24] 2.10.0+ was used to identify orthologous proteins from *Tetrahymena* mitochondria.

### 2.7. Generation of Injection Constructs

Artificial nanochromosome constructs were generated through overlap extension PCR using Phusion (New England Biolabs, Ipswich, MA, USA) as described previously [24]. A UAR = Q codon-corrected GFP was attached to the N-terminus of the protein including a linker sequence, and the artificial chromosome was cloned into *E. coli* TOP10 cells using a TOPO-TA cloning kit (Invitrogen, Waltham, MA, USA). Plasmids containing the chromosome were Sanger sequenced (Genewiz, South Plainfield, NJ, USA) for verification and the artificial chromosome was amplified using primers including the double-stranded portion of both telomeres. PCR products were ethanol precipitated and resuspended in nuclease-free water (Ambion, Austin, TX, USA) to a final concentration of >1 mg/mL, then filtered through a 0.2 micron centrifugal filter unit (Millipore, Burlington, MA, USA) before injection.

### 2.8. Transformation

Transformation of *Oxytricha trifallax* was performed as described previously [24]. Vegetatively growing JRB310 cells were starved overnight, then placed under a layer of mineral oil in a droplet of 0.2% *w/v* BSA in Volvic brand mineral water. The artificial chromosome solution was injected into the MAC of immobilized cells using a Narishige IM300 microinjector. Injected cells were placed into individual wells of a 24-well plate and allowed to expand clonally, passaging into 6-well plates and then 10 cm Petri dishes once cultures had been expanded enough. Positive transformants were identified through PCR of individual cells or DNA extracted using a NucleoSpin Tissue kit (Macherey-Nagel, Düren, Germany).

### 2.9. Western Blotting

Western blotting was performed on separated nuclear samples using a rabbit polyclonal antibody against GFP (Abcam ab6556, Cambridge, UK). Samples were lysed in RIPA buffer and boiled in Laemmli sample buffer for 10 min, then separated on an SDS-PAGE gel. Proteins were transferred to a PVDF membrane using a Trans-Blot SD semi-dry transfer apparatus (Bio-Rad, Hercules, CA, USA). Membranes were blocked with 4% nonfat dry milk in TBST and incubated with primary antibody overnight at 4 °C. Membranes were subsequently incubated with goat anti-rabbit antibody conjugated to horseradish peroxidase and imaged on an Amersham AI600RGB imager with ECL reagent.

### 2.10. Generation of Custom Antibodies

*Oxytricha trifallax* TEBP-α (Contig22209.0.g28473) was expressed and purified in BL21(DE3) cells as described previously [25]. Cells were cultured to OD = 0.6 in 2xYT medium and induced with 0.5 mM IPTG at 25 °C for 12 h. Induced cells were resuspended in lysis buffer (50 mM HEPES pH 7.5, 50 mM NaCl, 1 mM EDTA, 0.02%(*w/v*) sodium azide, 2 mM DTT) and sonicated. The lysate was clarified by centrifugation at 30,000× *g* for 30 min at 4 °C, and then subjected to ammonium sulfate fractionation: 20 g (NH_4_)_2_CO_3_ was added per 100 g of lysate, stirred on ice, then centrifuged at 30,000× *g* for 30 min at 4 °C. The cleared supernatant was transferred to a fresh tube, to which another 20 g of (NH_4_)_2_CO_3_ was added per 100 g of sample. After stirring on ice, the mixture was centrifuged at 30,000× *g* for 30 min at 4 °C and the supernatant was now discarded. The pellet was then dissolved in 15 mL of buffer A (25 mM HEPES pH 7.5, 100 mM NaCl, 0.25 mM EDTA, 0.02% (*w/v*) sodium azide, 1 mM DTT) and dialyzed into 1 liter of buffer A at 4 °C overnight. The dialyzed protein was further purified over a HiTrap SP HP column (Cytiva) and a Superdex 75 column (Cytiva). The Superdex 200 column was pre-equilibriated in (10 mM Tris pH 7.5, 50 mM NaCl, 0.1 mM EDTA, 0.02% (*w/v*) sodium azide, 1 mM DTT). Fractions containing TEBP-α were pooled and concentrated to 5–10 mg/mL at stored at 4 °C before further use. Polyclonal antibodies were generated against purified TEBP-α using the Mighty Quick protocol (Pocono Rabbit Farm & Laboratory, Canadensis, PA, USA).

Codon optimized Ku80 (Contig9679.0.g10501) from *Oxytricha trifallax* was cloned into a pET-28 vector with cleavable N-terminal poly-histidine tags (Novagen, Darmstadt, Germany) and expressed in *E. coli* BL21(DE3) cells. The expressed recombinant proteins were purified by affinity chromatography using His-Trap^TM^ HP columns (GE Healthcare) in PBS buffer containing 1 M NaCl and 0.2 mM TCEP (buffer A). Proteins were eluted with imidazole. Purified Ku80 fractions were digested with thrombin and dialyzed overnight against 100 volumes of buffer A. Digested samples were subjected to a second Ni^2+^ affinity purification step. The flow-through was collected and subjected to size-exclusion chromatography in a Superdex 200 10/300 column (GE Healthcare) previously equilibrated in 20 mM Tris pH 7.6, 200 mM NaCl and 0.2 mM TCEP. All purification steps were conducted at 4 °C. Antibodies were raised in rabbit hosts at Pocono Rabbit Farm and Laboratory.

### 2.11. Cell Imaging

Cells expressing the GFP-histone fusion protein were fixed in 4% paraformaldehyde and counterstained with DAPI to visualize the nuclei. The fixed cells were imaged using a Zeiss Axiovert 200 inverted fluorescence microscope. Immunofluorescent staining was performed as described previously [26] on vegetative JRB310 cells using the custom Ku80 and TEBP-α rabbit antibodies, anti-H3K14ac (Millipore), anti-H3K4me2 (Schedl Lab, Princeton, NJ, USA), or anti-H3K4me3 (Millipore) as the primary antibody. Images were captured using a Nikon A1 laser scanning confocal in the Confocal and Specialized Microscopy Shared Resource of the Herbert Irving Comprehensive Cancer Center at Columbia University.

## 3. Results

### 3.1. Oxytricha Nuclei Contain Distinct Proteomes

We cultured *Oxytricha* cells asexually and used sucrose gradient centrifugation to gather samples enriched in either MAC or MIC. We cold-starved the cells prior to this procedure, keeping the cells at 4 °C for up to 3 days after feeding to ensure that they fully digest the algae present within their food vacuoles to minimize algal contamination. This also helps prevent cells which have finished digesting their food from entering the starvation-induced encystment stage of their life cycle, which is resistant to the lysis buffer used in the nuclear isolation procedure. While this method was originally developed for DNA isolation and typically includes a secondary size-based separation afterwards [27,28], nuclear proteins do not share the same size bias between the MAC and MIC that chromosomes do. Despite this lack of secondary enrichment, MAC and MIC samples are noticeably different when separated on an SDS-PAGE gel, as well as when blotted with an anti-H3 histone antibody.

**Figure 1:**
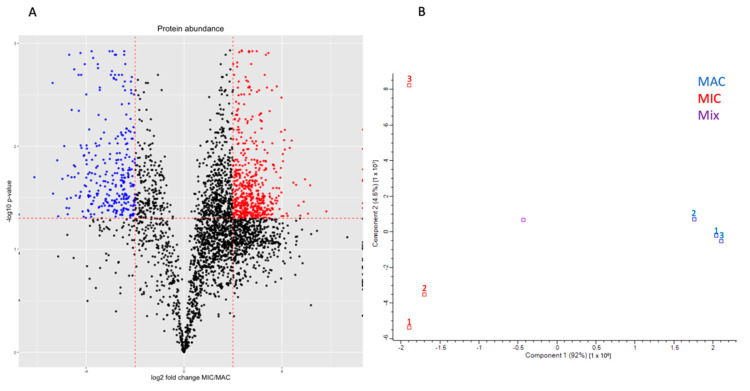
Proteomic analysis of TMT-labeled *Oxytricha* nuclear samples. (**A**) Volcano plot generated with ggplot2 [29] of identified protein groups from mass spectrometry data. Proteins predicted to localize to mitochondria using DeepLoc2 were removed. The log2 ratio of average MIC sample reporter ion intensity over average MIC sample reporter ion intensity was plotted against the inverse log10 of the FDR-adjusted *p*-value of the difference between nuclei. Proteins significantly enriched in either the MAC or MIC (log2 ratio < −2 or > 2 and adjusted *p*-value < 0.05) were plotted in blue and red points respectively. (**B**) PCA analysis generated with Perseus [30] of the intensities from the 3 MAC and MIC replicates are plotted in blue and red, respectively, as well as the control mix of all 6 samples plotted in purple. The MAC and MIC samples are well separated along PC1, which accounts for most of the variability between nuclear samples. While the MIC replicates do not cluster together as tightly as the MAC replicates on PC2, this component represents far less of the variance among the samples compared to PC1.

To identify the proteins that constitute the MAC and MIC, we prepared 3 replicates of nuclear samples and used tandem mass tag (TMT) labeling to quantitatively analyze the nuclear proteomes using LC-MS/MS at the MS3 level with MaxQuant. We analyzed the results using R, removing primary hits from *Oxytricha*’s food sources *Chlamydomonas reinhardtii* and *Klebsiella pneumoniae*. We identified 3644 proteins in this dataset, of which 341 are enriched in the MAC and 1277 in the MIC, with a fold-change of at least 2. With an adjusted *p*-value cutoff of 0.05, we find 285 significantly enriched MAC proteins and 587 MIC proteins (Figure 1).

### 3.2. Micronuclear Fraction Also Includes Other Organelles

We used DeepLoc 2.0 to predict the subcellular localization of all *Oxytricha* proteins identified from the mass spectrometry results. Of the 3644 proteins identified from our samples, 1434 were predicted to be nuclear. However, many of the MIC-enriched proteins were not predicted to be nuclear. Nearly all the predicted mitochondrial proteins were enriched in the MIC fraction, suggesting that mitochondria and potentially other organelles such as peroxisomes, lysosomes, and the endoplasmic reticulum tend to migrate in a similar pattern to micronuclei when separated using the previously established discontinuous sucrose gradients. Mitochondrial-predicted proteins from DeepLoc 2.0 were removed from further analyses, reducing the number of proteins to 3227 (Supplemental Table S1). The removal of the mitochondrial proteins resulted in a reduction of significantly enriched MIC proteins to 552 (Supplemental Table S2), while the number of significantly enriched MAC proteins was unchanged (Supplemental Table S3). The predicted mitochondrial proteins are listed in Supplemental Table S4.

The DeepLoc analysis predicted that 417 identified proteins were mitochondrial. A previous study of the proteomic composition of *Tetrahymena thermophila* mitochondria identified a similar number of proteins [31], with 563 proteins encoded from both the MAC and the mitochondrial genome. While this number is greater than those found in the MIC fraction, the enrichment of mitochondria in that study would have led to the identification of more proteins from their organelle of interest. Proteins from the *Oxytricha* mitochondrial genome were not included in our initial search, but a separate search against the proteins encoded by the *Oxytricha* mitochondrial genome identified peptides from 8 proteins, 6 of which were also found in *Tetrahymena*. Using BLASTP, we find that 230 of the predicted 418 mitochondrial proteins are homologous to the set of *Tetrahymena* mitochondrial proteins, providing further confidence in their mitochondrial origin. The set of genes encoded by the *Oxytricha* and *Tetrahymena* mitochondrial genomes are nearly identical [32,33], suggesting that a roughly equal number of mitochondrially-encoded genes may be present between the two species.

Previously, this technique provided adequate enrichment of the *Oxytricha* MIC genome for DNA sequencing, but any reads could be computationally filtered against the mitochondrial genome sequence [32]. Hence, while this technique can effectively separate the nuclei, it does not appear to be capable of separating the MIC from other small organelles. Studies in *Paramecium* have successfully demonstrated that flow cytometry may provide a better alternative to specifically isolate the two types of nuclei [34,35].

### 3.3. Distinct Functional Differences between MAC and MIC Proteins

**Figure 2:**
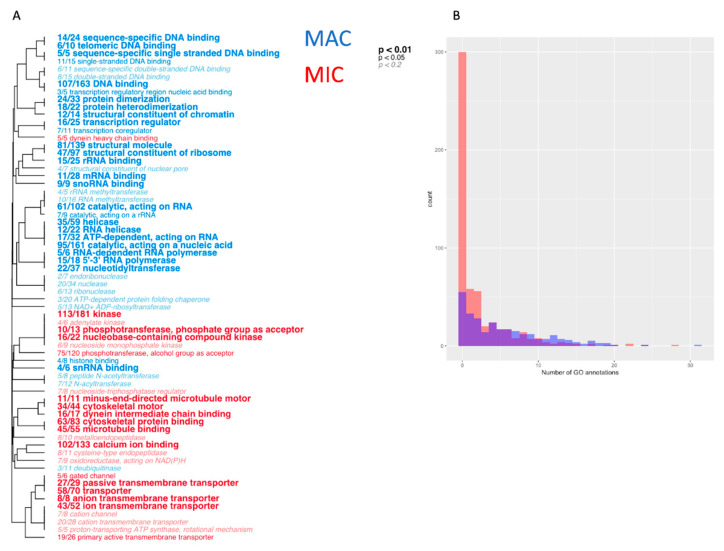
(**A**) GO term enrichment analysis of the nuclear samples. Terms significantly enriched in either the MAC or MIC are colored blue or red, respectively, using the Mann-Whitney U test and generated using the GO-MWU package [23]. (**B**) Histogram of the number of annotated GO terms for significantly enriched MAC and MIC proteins. A large proportion of MIC proteins lack any predicted terms.

To distinguish the functions of proteins identified in MAC and MIC fractions, we used Gene Ontology (GO) term enrichment analysis to test for differences of functional categories between nuclear samples. We used the GO-MWU package to analyze all the proteins identified in our data excluding mitochondrial proteins, using the log2 fold change between average MAC and MIC intensity as the measure of significance. The GO annotations for the proteins were generated by combining predictions from InterProScan and the Pannzer2 web server. Despite the combination of multiple sets of terms, there remains a large subset of identified proteins that were unable to have any GO categories assigned to them. MIC-enriched proteins tend to have far fewer terms than those in the MAC, with the most significantly enriched MIC proteins having no annotations at all, which limits the ability of this analysis to infer MIC-specific functions (Figure 2).

### 3.4. Different Sets of Core Histones Are Present in Respective Nuclei

**Figure 3:**
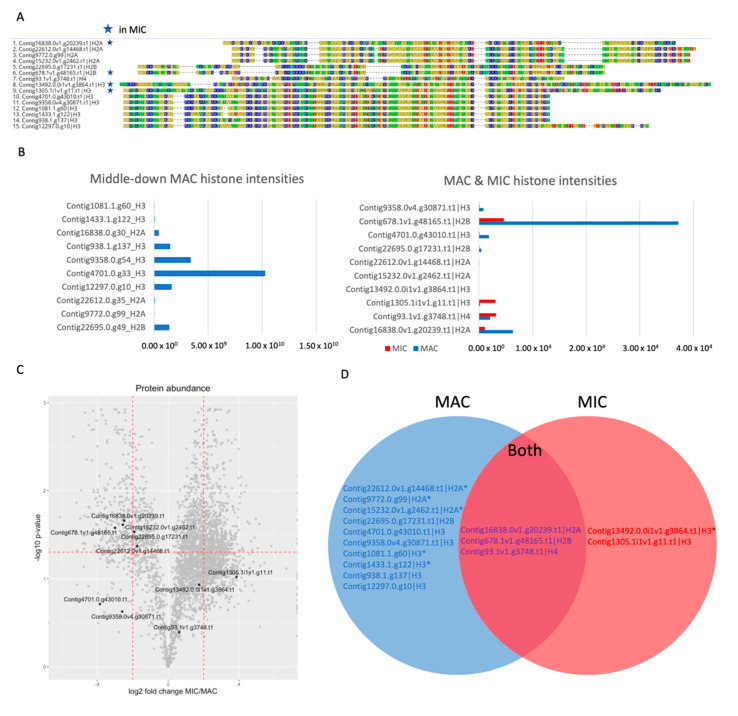
Differences in histone composition between MAC and MIC. (**A**) Alignment of all histones identified in both MS datasets. The first 425 residues and last 60 residues of Contig13492.0.0i1v1.g3864.t1 were omitted. Histones that are present in the MIC are starred. (**B**) Overall ion intensities (AU) for H2A, H2B, and H3 variants found in the middle-down MAC histones and separated nuclear samples. The middle-down dataset identified more histone variants than the nuclear dataset, likely due to the differences in sample preparation. (**C**) Volcano plot marking all the histones from the nuclear MS dataset. While only the MIC-specific H3 variants appear in the MIC-enriched fraction, there is a single H2A and H2B variant that has a high overall ion intensity from the MIC samples. (**D**) Venn diagram of histone presence among the two nuclei. Histones marked with an asterisk are present at low levels.

The complement of histone variants in each nucleus is known to be different in other ciliate species [36]. Our data shows that *Oxytricha trifallax* nuclei also have differences in histone composition, confirming previous observations of gel-separated acid-soluble nuclear proteins in a similar *Oxytricha* species. We identified a total of 3 H2A, 2 H2B, 4 H3, and 1 H4 histone among all nuclei (Figure 3). Previous studies on related *Oxytricha* species have not clearly identified the presence or absence of H1 linker histones in nuclear extracts [8,37]. While more recent reports suggest that the *Oxytricha* genome does encode two proteins with homology to H1 domains [38], we only identified one of them which appears to be enriched in the MIC. While linker H1 histones have been well-characterized in *Tetrahymena* [39], neither the MAC nor MIC *Tetrahymena* H1 variants appears to be homologous to any histone variants in *Oxytricha.* The MAC fraction has at least 1 variant of core histones H2A, H2B, and H3 enriched over the MIC. The MIC fraction is enriched in two H3 variants, but not in any other core histones. Despite the lack of enrichment of specific H2A or H2B variants, there does appear to be one of each that is present in the MIC at lower levels than the MAC based on reporter ion intensities. Both nuclei also appear to use the same H4 histone, as it is not significantly enriched within either fraction. The *Oxytricha* MAC genome only encodes a single H4 variant which explains its shared nature. These results may not fully represent the histone landscape of the *Oxytricha* micronucleus, as this experiment was performed using trypsin as the digestion enzyme, which can result in underrepresentation of certain histone peptides, due to their high lysine and arginine content.

### 3.5. MAC Histones Are Modified with Active Marks 

**Figure 4:**
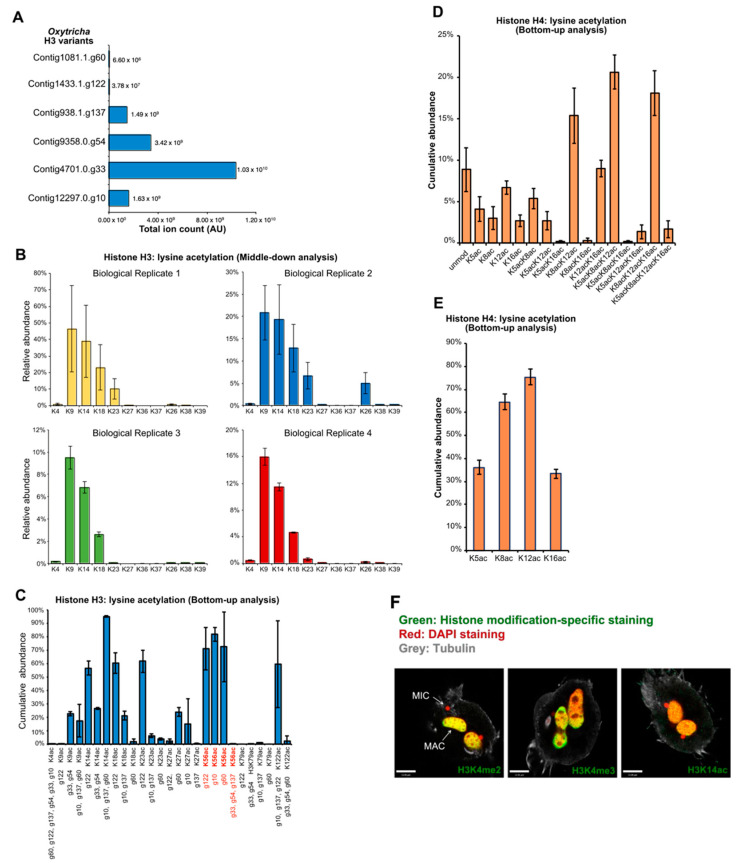
Detection of poly-acH4 and H3K56ac in *Oxytricha* cells using quantitative mass spectrometry. (**A**) Middle-down mass spectrometry (MS) quantification of histone H3 variants. Histone H3 variants are listed along the *y*-axis, and are henceforth abbreviated as g60, g122, g137, g54, g33, and g10 respectively. *x*-axis represents total ion count (arbitrary units). (**B**) Middle-down MS quantification of histone H3 (Contig4701.0.g33) acetylation. Data from four biological replicates are respectively shown. Positions of PTMs are listed along the *x*-axis. *y*-axis represents the cumulative abundance of acetylation on the modified Lys-residues as relative to the total histone H3. Each bar represents the averaged relative abundance (%) of 3 technical replicates (with exception of n_t_Rep4_ = 2); error bars represent ± standard deviation (stdev) of technical (n_t_Rep1-3_ = 3; n_t_Rep4_ = 2;) replicates. (**C**) Bottom-up MS quantification of histone H3 acetylation. Positions of PTMs are listed along the *x*-axis. *y*-axis represents the cumulative abundance of acetylation on each residue. Histone peptides containing H3K56ac are KYQKSTELLIR (g122); KFQKSTELLIR (g10); KYQKSTDLLIR (g60); and RFQKSTELLIR (g33, g54, g137). Each bar represents the averaged relative abundance (%) of 4 biological replicates; n_b_ = 4). Error bars represent ± standard deviation (stdev) of biological replicates (n_b_ = 4). (**D**) Bottom-up MS quantification of histone H4 acetylation. Positions of PTMs are listed along the *x*-axis. *y*-axis represents the cumulative abundance of acetylation on the four modified residues of the H4 peptide GKVGKGYGKVGAKR. The *Oxytricha* genome contains two annotated histone H4 genes with identical amino acid sequence. (**E**) Modified peptides from (**D**) as relative to total histone H4. Each bar represents the averaged relative abundance (%) of 4 biological replicates; n_b_ = 4). Error bars represent ± standard deviation (stdev) of biological (n_b_ = 4) replicates. (**F**) Immunofluorescence imaging of “active” H3 PTMs in vegetative cells.

To gain a quantitative picture of histone content in *Oxytricha*, we isolated vegetative MACs and solubilized histones by acid extraction. Histone H3 and H4 were further purified by reverse phase HPLC and analyzed by a middle-down and bottom-up proteomic workflow. Different histone H3 variants are detected, suggesting a role in marking regions of the macronuclear genome for different functions (Figure 4A). Notably, both middle-down and bottom-up analysis revealed abundant acetylation of the N-terminal tails of histone H3 and H4, including lysine 9, 14, 18, and 23 in H3, and lysine 5, 8, 12, and 16 in H4 (Figure 4B–E). Polyacetylation of H4 peptides is also observed, such as H4K5/8/12ac, H4K8/12/16ac, and H4K5/8/12/16ac (Figure 4D). Since these modifications are hallmarks of transcriptional activity in eukaryotes, our findings are consistent with previous reports indicating that the vegetative MAC is a hub of active chromatin [12,40]. Immunostaining for the “activating” histone modifications H3K4me3 and H3K14ac in the highly conserved N-terminal region of the MAC H3 histone variants showed specific localization to the MAC (Figure 4F), further supporting this notion. Lysine acetylation of histone H3 and H4 tails is a universally conserved feature, spanning metazoa, fungi, plants, and unicellular organisms in the SAR supergroup [41]. It is likely that *Oxytricha* uses canonical machinery for gene regulation, as in other eukaryotes. Indeed, homologs of the H3/H4 histone acetyltransferases Gcn5, Hat1, and NuA4, are readily identified in the *Oxytricha* MAC genome (Supplemental Table S5), and one of these Gcn5 homologs is present in our nuclear mass spectrometry dataset. These enzymes are conserved across eukaryotes, including *Tetrahymena*, yeast, plants, and animals [42]. They are likely to be responsible for the extensive H3 and H4 histone acetylation observed in *Oxytricha* MAC histones.

Nearly all the histones identified as MAC-enriched in the whole nuclear samples were also found in the middle-down samples, in which the proteins were digested using GluC instead of trypsin. The different digestion method also allowed for the identification of 3 more H3 variants that could not be distinguished from the predominant variant in the whole nuclear samples due to sequence similarity. These data were generated by searching against the predicted proteins from the previous *Oxytricha* MAC genome assembly [6]. While the corresponding proteins between the two assemblies do not have any differences in sequence, one of the H2A variants in the original assembly was collapsed into a different gene with identical sequence in the new assembly, resulting in a change in the gene name (Contig9772.0.g99 = Contig15232.0v1.g2462.t1). The predominant H2B variant appears to be different between the two datasets, but this is likely due to sequence similarity. These variants only differ in their N-terminal regions which are enriched in lysine and arginine residues, making it difficult to distinguish between the two proteins at the peptide level when digested with trypsin. 

### 3.6. RNA Polymerase Localizes Exclusively to the MAC

In ciliates, the MAC is the only nucleus that is transcriptionally active during vegetative growth [4,5]. Our results confirm that RNA polymerase subunits are greatly enriched in the MAC samples, with very little presence in the MIC samples. The only RNA polymerase proteins that are enriched in the MIC sample are predicted MAC-encoded mitochondrial polymerases, which is due to the presence of mitochondrial contamination within the MIC-enriched fraction. The MAC-specific presence of RNA polymerase components confirms previous work from the lab which demonstrated that the RPB1 subunit of the RNA polymerase II complex is exclusively found within the MAC in vegetative cells [43]. This is also in agreement with our observations that MAC chromatin is highly enriched in active PTMs.

### 3.7. Histone H3 Variant H3.8 Exclusively Localizes to the MIC

**Figure 5:**
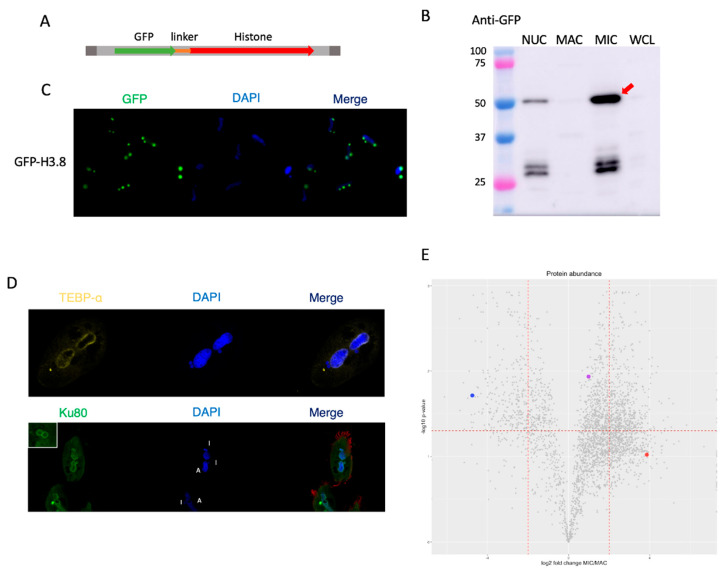
Validation of nuclear localization results from MS. (**A**) Schematic of the artificial chromosome used to transform *Oxytricha* with an N-terminal GFP-tagged H3 histone. A ciliate codon-corrected GFP was inserted at the N-terminus of the gene along with a flexible linker. The double-stranded portion of the nanochromosome’s telomeres were included at both ends. (**B**) Western blot analysis of samples from cells transformed with the previously described construct including unseparated nuclei (NUC), enriched MAC, enriched MIC, and whole cell lysate (WCL) using an anti-GFP antibody. The expected band of approximately 48 kDa is marked with an arrow. The smaller bands between 25 and 37 kDa are close to the size of free GFP (27 kDa). (**C**) Fluorescence imaging of the GFP-tagged strain depicting the specific MIC localization of the transgene with DAPI to visualize the nuclei. (**D**) Immunofluorescence imaging of vegetative JRB310 cells using custom antibodies raised against *Oxytricha* TEBP-α or Ku80, with DAPI to visualize nuclei demonstrating the MAC-specific localization of TEBP-α and the nonspecific nuclear localization of Ku80. (**E**) Volcano plot with the 3 validated proteins depicted as colored spots. TEBP-α is labeled in blue, Ku80 in purple, and H3.8 in red.

Previous studies in the closely-related ciliate *Stylonychia lemnae* indirectly identified a histone H3 variant that is only present within the MIC, along with its ortholog in *Oxytricha trifallax* [35,44]. We confirmed its MIC-specific localization by generating a transformed line expressing a GFP-tagged copy of histone H3.8 (Figure 5A–C). We observe green fluorescence exclusively in all copies of the MIC within these cells. Western blotting against separated nuclear samples of this line using an anti-GFP antibody also shows that GFP is only present within the MIC-enriched fraction. The results of our mass spectrometry data also support the specific MIC localization of this histone, which we found to be greatly enriched in the MIC samples (Figure 5E). While its *p*-value was above our significance cutoff, the MIC intensities were all an order of magnitude higher than in the MAC samples. 

### 3.8. Telomere End-Binding Protein Alpha Is Exclusively Localized to the MAC

The MAC telomeres of *Oxytricha* are known to be protected by a heterodimeric protein complex consisting of telomere end-binding protein (TEBP) α and β [45,46]. These proteins are found in high abundance in the MAC, as the highly polyploid nature of the tens of thousands of nanochromosomes found within the nucleus entails the presence of an enormous number of telomeres. While MIC chromosomes also have telomeres at their ends, they are far longer than those of the MAC, which have a distinctly defined 20 bp double-stranded segment with a 16-base single-stranded end [47]. We used a custom antibody raised against recombinant TEBP-α in rabbits to perform immunofluorescent imaging in order to examine its subcellular localization in vegetative cells. In accord with the mass spectrometry results (Figure 5E), TEBP-α only appears to be present within the vegetative MAC (Figure 5D).

### 3.9. Ku80 Is Present within Both Nuclei

The Ku heterodimer is a complex composed of two subunits, Ku70 and Ku80, that binds double-strand breaks in DNA and is an important factor in the non-homologous end joining pathway for DNA repair [48], as well as having roles in telomere maintenance [49]. Studies in other ciliates have shown that Ku80 is required for sexual development in *Tetrahymena* and *Paramecium* [50,51]. We observed that Ku80 is present within both types of nuclei in *Oxytricha* vegetative cells (Figure 5D), confirming the mass spectrometry results showing that it is present at roughly equal proportions in both types of nuclei (Figure 5E).

### 3.10. MIC-Enriched Domains

Many of the significantly enriched proteins in the MIC fraction have unknown function, annotated as hypothetical proteins. However, the organellar contamination from non-MIC particles in these samples makes it difficult to identify domains that are truly nuclear. A large proportion of these proteins are predicted to relate to ciliary structure and function, including cytoskeletal proteins, calcium ion binding domains, and centrosomal components [52]. While many of these proteins may not be nuclear, there are several proteins whose predicted functions are involved in DNA repair and replication, consistent with nuclear localization. There are many P-loop NTPases in the subset of significantly enriched MIC proteins. While this domain is commonly found in many different classes of proteins [53], the MIC proteins containing this domain include DNA-associated proteins such as ISWI, DNA helicases, and SMC complex proteins. The MIC is also enriched with multiple RCC1 proteins, which are involved with mitotic regulation [54] (Table 1).

### 3.11. MAC-Enriched Domains

The proteins significantly enriched in the MAC fraction are predominantly involved in nucleic acid production and maintenance, such as the previously mentioned RNA polymerase subunits (Table 2). Nucleolar and ribosomal biogenesis proteins are enriched as well, consistent with the presence of nucleoli only in the MAC. RNA-associated proteins, including helicases, splicing factors, and methyltransferases, are also abundant. Although transcriptional regulation in *Oxytricha* is not well understood, we find multiple transcription factor-related proteins within the MAC. The MAC also contains multiple bromodomain-containing proteins, which likely interact with the highly acetylated MAC histones [55]. Overall, the domains predominantly identified within the MAC are consistent with its state of active transcription.

## 4. Discussion

The paradigm of nuclear dualism in ciliates has long been a topic of great interest in the field of ciliate research. Here we performed a proteomic survey of both nuclei in a ciliate. Our results show that there are distinct compositional differences between the nuclei, and that many of the proteins that differ between the MAC and MIC are involved in the unique functions and features that distinguish them. 

While our method of nuclear separation yields sufficient purity for the MAC, our results demonstrate that the MIC fraction is less clean, containing many mitochondrial proteins in our samples. The difference in purity level between nuclear sample types is likely a result of the method used for enrichment, which was originally developed for analysis of MAC versus MIC DNA [13]. While it has proven adequate for nucleic acids, the extra organellar contamination in the MIC fraction is more pronounced with protein samples. The abundance of non-nuclear proteins within the MIC samples limits the usage of these data as a perfectly accurate depiction of MIC composition. Further evidence should be used to distinguish if proteins identified from the MIC fractions are truly present in the nucleus.

Functional analyses of the types of proteins enriched in the two samples revealed that many expected categories are specific to the MAC, particularly those involved in the maintenance of DNA and production of RNA and ribosomes. However, the MIC samples displayed overrepresentation of terms involved in mitochondrial function and ciliary structure. While this is partially due to the level of non-nuclear contamination within the MIC samples, it is also likely a result of the specialization of the MIC as an organelle. The ciliate MAC is more similar to a traditional eukaryotic nucleus than the MIC, sharing similar nuclear targeting signals and nuclear pore complex composition [10,56]. It has been demonstrated in *Tetrahymena* that while the MIC does have corresponding signals and structures, they differ from those found in other eukaryotes. If the MIC contains specialized proteins and complexes to maintain the germline genome, these may lack clear orthologs, which can convolute the annotation of ciliate MIC-specific genes. Even among ciliates, the difference in MIC genome architectures between *Oxytricha* and other commonly studied ciliates may require specialized proteins only found in other hypotrichs.

Consistent with previously known and observed information about ciliate nuclei, we find that RNA polymerase and transcription-related proteins are exclusively found within the MAC. The fact that the MIC is transcriptionally inactive during the vegetative life cycle has long been established, given the absence of RPB1 from the MIC, as well as the lack of RNA corresponding to MIC-limited sequences [26,39]. We also verified the active nature of the MAC through a separate mass spectrometry-based analysis of acid-extracted histones, displaying a great enrichment of activating acetylation marks among MAC H3 and H4 histones.

The histones identified from the whole nuclear samples also depicted a distinct difference in chromatin composition between the MAC and MIC. While the two nuclei appear to share common H2A, H2B, and H4 histones, the H3 variants were exclusive to either nucleus, and only a single putative H1 histone was identified in the MIC. This histone does not share homology with either of the well-characterized *Tetrahymena* linker histones [39], and its role in the nucleus is not known. The *Tetrahymena* MIC H1 is encoded as a single large precursor that is proteolytically processed into 3 smaller proteins and has been shown to be essential for chromosome maintenance during the vegetative stage, as well as for proper sexual development [57]. Given the drastic differences between the sequences of the *Tetrahymena* and *Oxytricha* MIC H1 variants, it is unclear if the *Oxytricha* MIC H1 histone plays a similar role in rearrangement as in *Tetrahymena.* We found two H3 variants in the MIC, both of which have much lower sequence similarity to canonical H3 histones. One of these MIC-specific H3 variants was previously speculated to localize exclusively to the MIC on the basis of orthology to a MIC-specific histone in *Stylonychia lemnae* [40]. By transforming vegetative cells with a GFP-tagged version of this gene, we confirmed its true MIC presence.

The other MIC-enriched histone H3 is less prevalent in the cell, having only a single peptide identified with low ion intensity. This variant is highly diverged from the rest of the identified H3 histones, sharing less than 50% amino acid identity with any other histone. Additionally, the predicted ORF for this protein includes extensive N and C terminal tails of over 400 and 100 amino acids, respectively. While these long extensions may be legitimate, it is likely that they are simply due to gene prediction inaccuracy, as the single peptide from this protein was from a region slightly downstream of the conserved histone fold domain but before the extended C-terminal tail, and its ortholog in *Stylonychia lemnae* also does not include either of the extended tails [35]. Given its relatively lower abundance than the other MIC H3, it is possible that this may serve as the centromeric H3 histone for *Oxytricha. Tetrahymena thermophila* [58] and *Paramecium tetraurelia* [59] both encode a CENP-A ortholog that is present in MIC centromeres and is required for proper mitotic division of the nucleus. No direct ortholog to CENP-A has been identified in *Oxytricha* or *Stylonychia*, though they both have MIC centromeres [40]. 

While the nuclei each have their own sets of H3 variants, the single H2A and H2B variants within the MIC are also found in the MAC. However, the MAC also contains other H2A and H2B variants along with the ones present in the MIC. It is possible that the shared histone variants may be used as a form of transcriptional regulation. While the MAC is generally actively transcribed, it contains genes that are not expressed during the vegetative life cycle [6]. These genes, along with certain chromosomes that do not appear to encode any genes [23], may share the MIC variant of these histones to ensure their transcriptional repression when unneeded, though further investigation is required to determine if this is the case. 

While the presence of the TEBPs in the MAC was expected, we curiously do not identify any TEBPs enriched in the MIC fraction, even though the MAC genome encodes six paralogs of TEBP-α and three paralogs of TEBP-β [6]. Only three of the TEBP-α paralogs and two of the TEBP-β paralogs appear in our dataset, including the primary variants TeBP-α1 and TeBP-β1. Thus, it is possible that some of the other paralogs are either too low in abundance or exclusively expressed during development. The antibody used in this study was raised against the original TEBP-α1, though we anticipate it may have some cross-reactivity with at least one of its paralogs. The primary TEBP complex is known to bind strongly to MAC telomeres [42], but their paralogs have not been well characterized. Since MIC telomeres contain longer tracts of the same sequence repeats as MAC telomeres [60], it has been hypothesized that different TEBP complexes may be specialized to telomeres of the different nuclei [6]. While all TEBP proteins identified were highly enriched in the MAC fraction, this does not necessarily preclude their presence in the MIC. The MAC, with its average ploidy ~2000 for over 18,000 chromosomes, contains tens of millions of distinct telomeres that are bound by the TEBP complex. The diploid MIC, on the other hand, is estimated to have on the order of 100 significantly longer telomeres [26] which is many orders of magnitude less than in the MAC. Given this incredible disparity of telomere copies between the MAC and MIC, any enrichment of TEBP proteins in the MAC may simply be due to the proportional difference in telomere number between the two nuclei.

The present study offers the first survey of both nuclear proteomes in a ciliate. By using *Oxytricha trifallax* as a model, with its extreme differences in MAC and MIC genome architectures, we identified many proteins specific to the MAC and a set of proteins that may be enriched in the MIC and these may serve specific roles in the maintenance of the two distinct genomes during the vegetative life cycle. Despite contamination of other organellar material within the MIC samples, these datasets will provide a valuable resource for further studies of nuclear proteins that may further our understanding of the unique nuclear architectures common to ciliates.

## Figures and Tables

**Table 1:** **InterPro domains found in MIC-enriched proteins**. Domains that were found in at least four proteins are listed, with redundant domain entries removed. The complete list is provided in Supplementary Table S6.

InterPro Domain	Count
EF-hand domain pair	36
WD40/YVTN repeat-like-containing domain superfamily	26
P-loop containing nucleoside triphosphate hydrolase	21
Leucine-rich repeat domain superfamily	17
Armadillo-type fold	13
Tetratricopeptide-like helical domain superfamily	13
Protein kinase-like domain superfamily	10
WD40 repeat, conserved site	10
Regulator of chromosome condensation, RCC1	9
C2 domain superfamily	6
GAR domain	6
Giardin subunit beta-like	6
Growth factor receptor cysteine-rich domain superfamily	6
Kinesin motor domain	6
Serine/threonine-protein kinase, active site	6
Transient receptor potential cation channel subfamily V	6
Band 7 domain	5
Ion transport domain	5
IQ motif, EF-hand binding site	5
Papain-like cysteine peptidase superfamily	5
Protein kinase, ATP-binding site	5
Quinoprotein alcohol dehydrogenase-like superfamily	5
14-3-3 domain	4
AAA+ ATPase domain	4
Ankyrin repeat-containing domain superfamily	4
B-box-type zinc finger	4
Enkurin domain	4
Kinesin-like protein	4
MORN motif	4
SAS-6, N-terminal domain superfamily	4
Zinc finger, RING/FYVE/PHD-type	4

**Table 2:** **InterPro domains found in MAC-enriched proteins**. Domains that were found in at least four proteins are listed, with redundant domain entries removed. The complete list is provided in Supplementary Table S7.

InterPro Domain	Count
WD40 repeat-containing domain superfamily	28
P-loop containing nucleoside triphosphate hydrolase	16
Nucleic acid-binding, OB-fold	13
Histone-fold	11
Homeobox-like domain superfamily	11
Nucleotide-binding alpha-beta plait domain superfamily	11
RNA-binding domain superfamily	11
Helicase, C-terminal	9
RNA recognition motif domain	9
DEAD/DEAH box helicase domain	8
Helicase superfamily 1/2, ATP-binding domain	8
RNA helicase, DEAD-box type, Q motif	7
SANT/Myb domain	7
Ankyrin repeat	6
Armadillo-type fold	6
LSM domain superfamily	6
S-adenosyl-L-methionine-dependent methyltransferase	6
Zinc finger, RING/FYVE/PHD-type	5
ATP-dependent RNA helicase DEAD-box, conserved site	4
K Homology domain, type 1 superfamily	4
Poly(ADP-ribose) polymerase, catalytic domain	4
Poly(ADP-ribose) polymerase, regulatory domain superfamily	4
Tetratricopeptide-like helical domain superfamily	4
WGR domain superfamily	4
Winged helix-like DNA-binding domain superfamily	4

## Data Availability

Raw mass spectrometry data from nuclear samples are available on MassIVE (https://massive.ucsd.edu/) under identifier MSV000090958 and raw data from MAC histone samples are available on the Chorus database (https://chorusproject.org/ (accessed on 1 February 2015)) under project 1298.

## Supplementary Materials

The following supporting information can be downloaded at: https://www.mdpi.com/article/10.3390/microorganisms11020343/s1, Table S1: All identified proteins from nuclear samples, Table S2: Proteins significantly enriched in MIC samples, Table S3: Proteins significantly enriched in MAC samples, Table S4: Predicted mitochondrial proteins, Table S5: Histone H3/H4 Histone Acetyltransferases identified in the *Oxytricha* MAC genome, Table S6: All InterPro domains detected from proteins significantly enriched in the MIC, Table S7: All InterPro domains detected from proteins significantly enriched in the MAC fraction.

## References

[1] Lamond A.I., Earnshaw W.C. (1998). Structure and function in the nucleus. Science.

[2] Bhat P., Honson D., Guttman M. (2021). Nuclear compartmentalization as a mechanism of quantitative control of gene expression. Nat. Rev. Mol. Cell. Biol..

[3] De Magistris P., Antonin W. (2018). The Dynamic Nature of the Nuclear Envelope. Curr. Biol..

[4] Prescott D.M. (1994). The DNA of ciliated protozoa. Microbiol. Rev..

[5] Bracht J.R., Fang W., Goldman A.D., Dolzhenko E., Stein E.M., Landweber L.F. (2013). Genomes on the edge: Programmed genome instability in ciliates. Cell.

[6] Swart E.C., Bracht J.R., Magrini V., Minx P., Chen X., Zhou Y., Khurana J.S., Goldman A.D., Nowacki M., Schotanus K. (2013). The *Oxytricha trifallax* macronuclear genome: A complex eukaryotic genome with 16,000 tiny chromosomes. PLoS Biol..

[7] Lindblad K.A., Pathmanathan J.S., Moreira S., Bracht J.R., Sebra R.P., Hutton E.R., Landweber L.F. (2019). Capture of complete ciliate chromosomes in single sequencing reads reveals widespread chromosome isoforms. BMC Genom..

[8] Caplan E.B. (1977). Histones and other basic nuclear proteins in genetically active and genetically inactive nuclei of the ciliate, *Oxytricha* sp. Biochim. Biophys. Acta.

[9] Zhang C., Gao S., Molascon A.J., Liu Y., Andrews P.C. (2014). Quantitative proteomics reveals histone modifications in crosstalk with H3 lysine 27 methylation. Mol. Cell. Proteom..

[10] Iwamoto M., Osakada H., Mori C., Fukuda Y., Nagao K., Obuse C., Hiraoka Y., Haraguchi T. (2017). Compositionally distinct nuclear pore complexes of functionally distinct dimorphic nuclei in the ciliate *Tetrahymena*. J. Cell Sci..

[11] Malone C.D., Falkowska K.A., Li A.Y., Galanti S.E., Kanuru R.C., Lamont E.G., Mazzarella K.C., Micev A.J., Osman M.M., Piotrowski N.K. (2008). Nucleus-specific importin alpha proteins and nucleoporins regulate protein import and nuclear division in the binucleate *Tetrahymena thermophila*. Eukaryot. Cell.

[12] Beh L.Y., Debelouchina G.T., Clay D.M., Thompson R.E., Lindblad K.A., Hutton E.R., Bracht J.R., Sebra R.P., Muir T.W., Landweber L.F. (2019). Identification of a DNA N6-Adenine Methyltransferase Complex and Its Impact on Chromatin Organization. Cell.

[13] Lauth M.R., Spear B.B., Heumann J., Prescott D.M. (1976). DNA of ciliated protozoa: DNA sequence diminution during macronuclear development of Oxytricha. Cell.

[14] Navarrete-Perea J., Yu Q., Gygi S.P., Paulo J.A. (2018). Streamlined Tandem Mass Tag (SL-TMT) Protocol: An Efficient Strategy for Quantitative (Phospho)proteome Profiling Using Tandem Mass Tag-Synchronous Precursor Selection-MS3. J. Proteome Res..

[15] Cox J., Mann M. (2008). MaxQuant enables high peptide identification rates, individualized p.p.b.-range mass accuracies and proteome-wide protein quantification. Nat. Biotechnol..

[16] (2022). R: A Language and Environment for Statistical Computing.

[17] (2020). RStudio: Integrated Development for R.

[18] Yuan Z., Lin S., Molden R.C., Cao X., Bhanu N.V., Wang X., Sidoli S., Liu S., Garcia B.A. (2015). EpiProfile Quantifies Histone Peptides with Modifications by Extracting Retention Time and Intensity in High-resolution Mass Spectra. Mol. Cell. Proteom..

[19] Sidoli S., Schwämmle V., Ruminowicz C., Hansen T.A., Wu X., Helin K., Jensen O.N. (2014). Middle-down hybrid chromatography/tandem mass spectrometry workflow for characterization of combinatorial post-translational modifications in histones. Proteomics.

[20] Jones P., Binns D., Chang H., Fraser M., Li W., McAnulla C., McWilliam H., Maslen J., Mitchell A., Nuka G. (2014). InterProScan 5: Genome-scale protein function classification. Bioinformatics.

[21] Törönen P., Medlar A., Holm L. (2018). PANNZER2: A rapid functional annotation web server. Nucleic Acids Res..

[22] Thumuluri V., Armenteros J.J.A., Johansen A.R., Nielsen H., Winther O. (2022). DeepLoc 2.0: Multi-label subcellular localization prediction using protein language models. Nucleic Acids Res..

[23] Wright R.M., Aglyamova G.V., Meyer E., Matz M.V. (2015). Gene expression associated with white syndromes in a reef building coral, *Acropora hyacinthus*. BMC Genom..

[24] Camacho C., Coulouris G., Avagyan V., Ma N., Papadopoulos J., Bealer K., Madden T.L. (2008). BLAST+: Architecture and applications. BMC Bioinform..

[25] Horvath M.P., Schweiker V.L., Bevilacqua J.M., Ruggles J.A., Schultz S.C. (1998). Crystal structure of the *Oxytricha nova* telomere end binding protein complexed with single strand DNA. Cell.

[26] Fang W., Wang X., Bracht J.R., Nowacki M., Landweber L.F. (2012). Piwi-interacting RNAs protect DNA against loss during *Oxytricha* genome rearrangement. Cell.

[27] Chen X., Bracht J.R., Goldman A.D., Dolzhenko E., Clay D.M., Swart E.C., Perlman D.H., Doak T.G., Stuart A., Amemiya C.T. (2014). The Architecture of a Scrambled Genome Reveals Massive Levels of Genomic Rearrangement during Development. Cell.

[28] Feng Y., Neme R., Beh L.Y., Chen X., Braun J., Lu M.W., Landweber L.F. (2022). Comparative genomics reveals insight into the evolutionary origin of massively scrambled genomes. eLife.

[29] Wickham H. (2016). ggplot2: Elegant Graphics for Data Analysis.

[30] Tyanova S., Temu T., Sinitcyn P., Carlson A., Hein M.Y., Geiger T., Mann M., Cox J. (2016). The Perseus computational platform for comprehensive analysis of (prote)omics data. Nat. Methods.

[31] Smith D.G.S., Gawryluk R.M.R., Spencer D.F., Pearlman R.E., Siu K.W.M., Gray M.W. (2007). Exploring the Mitochondrial Proteome of the Ciliate Protozoon *Tetrahymena thermophila*: Direct Analysis by Tandem Mass Spectrometry. J. Mol. Biol..

[32] Swart E.C., Nowacki M., Shum J., Stiles H., Higgins B.P., Doak T.G., Schonatus K., Magrini V.J., Minx P., Mardis E.R. (2012). The *Oxytricha trifallax* mitochondrial genome. Genome Biol. Evol..

[33] Brunk C.F., Lee L.C., Tran A.B., Li J. (2003). Complete sequence of the mitochondrial genome of *Tetrahymena thermophila* and comparative methods for identifying highly divergent genes. Nucleic Acids Res..

[34] Guérin F., Arnaiz O., Boggetto N., Wilkes C.D., Meyer E., Sperling L., Duharcourt S. (2017). Flow cytometry sorting of nuclei enables the first global characterization of *Paramecium* germline DNA and transposable elements. BMC Genom..

[35] Zangarelli C., Arnaiz O., Bourge M., Gorrichon K., Jaszczyszyn Y., Mathy N., Escoriza L., Betermier M., Regnier V. (2022). Developmental timing of programmed DNA elimination in *Paramecium tetraurelia* recapitulates germline transposon evolutionary dynamics. Genome Res..

[36] Schlegel M., Muller S., Ruder F., Büsen W. (1990). Transcriptionally inactive micronuclei, macronuclear anlagen and transcriptionally active macronuclei differ in histone composition in the hypotrichous ciliate *Stylonychia lemnae*. Chromosoma.

[37] Butler A.P., Laughlin T.J., Cadilla C.L., Henry J.M., Olins D.E. (1984). Physical structure of gene-sized chromatin from the protozoan *Oxytricha*. Nucleic Acids Res..

[38] Aeschlimann S.H., Jönsson F., Postberg J., Stover N.A., Petera R.L., Lipps H.J., Nowacki M., Swart E.C. (2014). The Draft Assembly of the Radically Organized *Stylonychia lemnae* Macronuclear Genome. Genome Biol. Evol..

[39] Nabeel-Shah S., Ashraf K., Saettone A., Garg J., Derynck J., Lambert J., Pearlman R.E., Fillingham J. (2020). Nucleus-specific linker histones Hho1 and Mlh1 form distinct protein interactions during growth, starvation and development in *Tetrahymena thermophila*. Sci. Rep..

[40] Postberg J., Heyse K., Cremer M., Cremer T., Lipps H.J. (2008). Spatial and temporal plasticity of chromatin during programmed DNA-reorganization in *Stylonychia* macronuclear development. Epigenetics Chromatin.

[41] Grau-Bové X., Navarrete C., Chiva C., Pribasnig T., Antó M., Torruella G., Galindo L.J., Lang B.F., Moreira D., López-Garcia P. (2022). A phylogenetic and proteomic reconstruction of eukaryotic chromatin evolution. Nat. Ecol. Evol..

[42] Wahab S., Saettone A., Nabeel-Shah S., Dannah N., Fillingham J. (2020). Exploring the Histone Acetylation Cycle in the Protozoan Model *Tetrahymena thermophila*. Front. Cell Dev. Biol..

[43] Khurana J.S., Wang X., Chen X., Perlman D.H., Landweber L.F. (2014). Transcription-independent functions of an RNA polymerase II subunit, Rpb2, during genome rearrangement in the ciliate, *Oxytricha trifallax*. Genetics.

[44] Forcob S., Bulic A., Jönsson F., Lipps H.J., Postberg J. (2014). Differential expression of histone H3 genes and selective association of the variant H3.7 with a specific sequence class in *Stylonychia* macronuclear development. Epigenetics Chromatin.

[45] Gottschling D.E., Zakian V.A. (1986). Telomere proteins: Specific recognition and protection of the natural termini of *Oxytricha* macronuclear DNA. Cell.

[46] Fang G., Cech T.R. (1993). *Oxytricha* telomere-binding protein: DNA-dependent dimerization of the alpha and beta subunits. Proc. Natl. Acad. Sci. USA.

[47] Klobutcher L.A., Swanton M.T., Donini P., Prescott D.M. (1981). All gene-sized DNA molecules in four species of hypotrichs have the same terminal sequence and an unusual 3’ terminus. Proc. Natl. Acad. Sci. USA.

[48] Walker J.R., Corpina R.A., Goldberg J. (2001). Structure of the Ku heterodimer bound to DNA and its implications for double-strand break repair. Nature.

[49] Fisher T.S., Zakian V.A. (2005). Ku: A multifunctional protein involved in telomere maintenance. DNA Repair.

[50] Lin I.T., Chao J.L., Yao M.C. (2012). An essential role for the DNA breakage-repair protein Ku80 in programmed DNA rearrangements in *Tetrahymena thermophila*. Mol. Biol. Cell.

[51] Abello A., Régnier V., Arnaiz O., Le Bars R., Bétermier M., Bischerour J. (2020). Functional diversification of *Paramecium* Ku80 paralogs safeguards genome integrity during precise programmed DNA elimination. PLoS Genet..

[52] Kilburn C.L., Pearson C.G., Romijn E.P., Meehl J.B., Giddings Jr T.H., Culver B.P., Yates J.R., Winey M. (2007). New Tetrahymena basal body protein components identify basal body domain structure. J. Cell Biol..

[53] Longo L.M., Jablonska J., Vyas P., Kanade M., Kolodny R., Ben-Tal N., Tawfik D.S. (2020). On the emergence of P-Loop NTPase and Rossmann enzymes from a Beta-Alpha-Beta ancestral fragment. eLife.

[54] Bischoff F.R., Ponstingl H. (1991). Mitotic regulator protein RCC1 is complexed with a nuclear ras-related polypeptide. Proc. Natl. Acad. Sci. USA.

[55] Jain A.K., Barton M.C. (2017). Bromodomain Histone Readers and Cancer. J. Mol. Biol..

[56] Iwamoto M., Mori C., Osakada H., Koujin T., Hiraoka Y., Haraguchi T. (2018). Nuclear localization signal targeting to macronucleus and micronucleus in binucleated ciliate *Tetrahymena thermophila*. Genes Cells.

[57] Qiao J., Xu J., Bo T., Wang W. (2017). Micronucleus-specific histone H1 is required for micronuclear chromosome integrity in *Tetrahymena thermophila*. PLoS ONE.

[58] Cervantes M.D., Xi X., Vermaak D., Yao M.C., Malik H.S. (2006). The CNA1 Histone of the Ciliate *Tetrahymena thermophila* is Essential for Chromosome Segregation in the Germline Micronucleus. Mol. Biol. Cell.

[59] Lhuillier-Akakpo M., Guérin F., Frapporti A., Duharcourt S. (2016). DNA deletion as a mechanism for developmentally programmed centromere loss. Nucleic Acids Res..

[60] Dawson D., Herrick G. (1984). Telomeric properties of C4A4-homologous sequences in micronuclear DNA of *Oxytricha fallax*. Cell.

